# Amino acids and RagD potentiate mTORC1 activation in CD8^+^ T cells to confer antitumor immunity

**DOI:** 10.1136/jitc-2020-002137

**Published:** 2021-04-21

**Authors:** Yiwen Zhang, Hongrong Hu, Weiwei Liu, Shu-Mei Yan, Yuzhuang Li, Likai Tan, Yingshi Chen, Jun Liu, Zhilin Peng, Yaochang Yuan, Wenjing Huang, Fei Yu, Xin He, Bo Li, Hui Zhang

**Affiliations:** 1 Institute of Human Virology, Key Laboratory of Tropical Disease Control of Ministry of Education, Guangdong Engineering Research Center for Antimicrobial Agent and Immunotechnology, Zhongshan School of Medicine, Sun Yat-sen University, Guangzhou, Guangdong, China; 2 Department of Biochemistry, Zhongshan School of Medicine, Sun Yat-sen University, Guangzhou, Guangdong, China; 3 Sun Yat-sen University Cancer Center, Sun Yat-sen University, Guangzhou, Guangdong, China; 4 Institute of Immunology, Hannover Medical School, Hannover, Niedersachsen, Germany; 5 School of Medicine, Sun Yat-sen University, Shenzhen, China; 6 Guangdong Provincial People's Hospital, Guangdong Academy of Medical Sciences, Guangzhou, Guangdong, China

**Keywords:** CD8-positive T-lymphocytes, lymphocytes, tumor-infiltrating, metabolic networks and pathways, lymphocyte activation, tumor microenvironment

## Abstract

**Background:**

In the tumor microenvironment, tumor cells are able to suppress antitumor immunity by competing for essential nutrients, including amino acids. However, whether amino acid depletion modulates the activity of CD8^+^ tumor-infiltrating lymphocytes (TILs) is unclear.

**Method:**

In this study, we evaluated the roles of amino acids and the Rag complex in regulating mammalian target of rapamycin complex 1 (mTORC1) signaling in CD8^+^ TILs.

**Results:**

We discovered that the Rag complex, particularly RagD, was crucial for CD8^+^ T-cell antitumor immunity. RagD expression was positively correlated with the antitumor response of CD8^+^ TILs in both murine syngeneic tumor xenografts and clinical human colon cancer samples. On RagD deficiency, CD8^+^ T cells were rendered more dysfunctional, as demonstrated by attenuation of mTORC1 signaling and reductions in proliferation and cytokine secretion. Amino acids maintained RagD-mediated mTORC1 translocation to the lysosome, thereby achieving maximal mTORC1 activity in CD8^+^ T cells. Moreover, the limited T-cell access to leucine (LEU), overshadowed by tumor cell amino acid consumption, led to impaired RagD-dependent mTORC1 activity. Finally, combined with antiprogrammed cell death protein 1 antibody, LEU supplementation improved T-cell immunity in MC38 tumor-bearing mice in vivo.

**Conclusion:**

Our results revealed that robust signaling of amino acids by RagD and downstream mTORC1 signaling were crucial for T-cell receptor-initiated antitumor immunity. The characterization the role of RagD and LEU in nutrient mTORC1 signaling in TILs might suggest potential therapeutic strategies based on the manipulation of RagD and its upstream pathway.

## Introduction

The unprecedented clinical outcomes achieved by cancer immunotherapy have revealed the potential applications of harnessing immunity to fight cancer.^1 2^ However, the therapeutic efficacy of these treatments has been hindered by therapeutic resistance because of dysfunctions in CD8^+^ tumor-infiltrating lymphocytes (TILs).^3 4^ In most established tumors, CD8^+^ T cells progressively lose their cardinal effector capacities, as exemplified by limited cytokine secretion and reduced proliferative capacity.^3 4^ Furthermore, these cells show high expression of immune checkpoint receptors and exhibit distinct transcriptional profiles and unique epigenetic landscapes.^5^ This restricted antitumor response is related to multiple environmental factors, including persistent antigen exposure and metabolic transformation in the tumor microenvironment (TME).^4 6 7^ Increasing evidence suggests that nutrient availability in the TME is a key factor affecting the immune response.^8 9^ For example, the limited supply of glucose leads to a decrease in aerobic glycolysis in TILs, thereby impairing T-cell receptor (TCR) activation, elevating lactate production by tumor cells and inhibiting TIL proliferation and cytokine production.^10–12^ Although recent studies have explored how tumor cells disrupt methionine metabolism in CD8^+^ TILs, the roles of amino acids in antitumor immunity remain elusive.^13^

Amino acids are important molecules that modulate the proliferative drive of tumor cells and have direct roles as protein synthesis substrates. Amino acids also function in energy metabolism, maintenance of cellular redox homeostasis and epigenetic modification. Interestingly, on TCR activation, CD8^+^ lymphocytes undergo a metabolic switch similar to that of cancer cells and upregulate glycolysis to meet the demand for proliferation and differentiation into specialized functional effector T cells.^14^ This reprogramming process also consumes large amounts of amino acids, as well as glucose and fatty acids.^15–17^ Furthermore, to induce T-cell proliferation and metabolic programs, TCR signals must meet a certain threshold of activation, as determined by the activation level of mammalian target of rapamycin (mTOR) complex 1 (mTORC1).^18^ Given the similarities in metabolic characteristics and nutrient requirements, it is possible that the abnormally high metabolic rates and consumption of amino acids by tumor cells compete with neighboring T cells, leading to T-cell metabolic dysfunction. Therefore, restriction of amino acid availability could be an environmental limitation that causes T-cell dysfunction. It is important to elucidate whether and how this affects TCR strength and T-cell function in the TME. Characterization of the amino acid sensing and metabolic functions of CD8^+^ TILs could provide new insights into therapeutic interventions aimed at enhancing T-cell functional fitness.^19^

mTOR is a key integrator of environmental cues to ensure that nutrient and growth factors are favorable for cell proliferation.^18^ The mechanisms through which amino acid availability affects the activation of mTORC1 may be related to the roles of Ras-related GTPase (Rag).^20^ Mammals express four Rag proteins, that is, RagA–D. RagA and RagB form heterodimers with RagC and RagD.^20^ On amino acid stimulation, the active Rag complex, consisting of RagA/B in the guanosine triphosphate (GTP)-bound state and RagC/D in the guanosine diphosphate-bound state, promotes mTORC1 translocation to the lysosome for maximal activation.^21^ RagD can interact with the C-terminal region of leucyl-tRNA synthase (LRS), which acts as a direct sensor for the amino acid leucine (LEU) and is involved in the activation of mTORC1.^22 23^ Recent studies have demonstrated the indispensable roles of nutrient sensing though the RagA protein in orchestrating embryonic development, mediating hematopoietic stem cell function and promoting regulatory T-cell (Treg) functions.^24–26^ However, the function of the Rag complex in CD8^+^ T-cell responses, particularly in the TME, have not been fully elucidated.

In this study, we assessed whether amino acid signals were regulated by RagD to potentiate TCR-mediated mTORC1 signaling in CD8^+^ T cells, particularly with regard to mTORC1 activation and the antitumor functions of CD8^+^ TILs in the TME. Moreover, we evaluated the roles of LEU in sustaining mTORC1 activity in CD8^+^ T-cells and examined the effects of abnormal LEU metabolism on mTORC1 activity in T cells and the TME. LEU supplementation improved T cell immunity in MC38 tumor-bearing mice in vivo. Our data supported that RagD-dependent amino acids regulated TCR-induced mTORC1 activation to modulate CD8^+^ T-cell antitumor immune responses.

## Materials and methods

### Ethics statement

Unidentified human peripheral blood mononuclear cells (PBMCs) of healthy blood donors were provided by the Guangzhou Blood Center. We did not have any interaction with these human subjects or protected information, and therefore no informed consent was required.

### Cell lines and mice

The HEK-293T cell line was obtained from the American Type Culture Collection. The B16-OVA cell line was gift from Professor Zhaofeng Huang of Sun Yat-sen University. MC38 and MC38-OVA cell line were gifts from Professor Penghui Zhou of Sun Yat-sen University. Mycoplasma testing was carried out by direct culture and Hoechst DNA staining and Limulus amoebocyte lysate assay to measure endotoxin values. C57BL/6, OT-I, *Cd4*^Cre^, CD45.1 and *Rag1*^−/−^ were purchased from GemPharmatech. *Rragd^fl/fl^* mice was generated and purchased from Cyagen. We crossed *Rragd^fl/fl^* mice with OT-I and *Cd4*^Cre^ and to generate antigen-specific CD8^+^ T cells. Sex-matched mice aged 6–8 weeks were compared with littermate controls.

### Medium preparation

Amino acid-deficient and amino acid-sufficient medium were homemade. Amino acid-free (AA–) medium was prepared using RPMI 1640 powder (R8999-04A, US Biological Life Science) and sodium phosphate dibasic (5.6 mM, the same concentration as commercially available RPMI 1640 medium, GIBCO) and supplemented with 10% dialyzed fetal bovine serum (FBS) (GIBCO). Amino acid-sufficient (AA+) medium was prepared by adding proper volumes of MEM amino acids solution (essential amino acids (EAA), 50×), MEM non-essential amino acids solution (NEAA, 100×) and 200 mM L-Gln (GIBCO). The medium was supplemented with 10% dialyzed FBS. Medium containing single amino acid (LEU or arginine (ARG)) was prepared with AA– medium (prepared to the same concentrations present in the AA+ medium), while medium deficient for single amino acid was prepared by adding all the EAAs or NEAAs to AA– medium, excluding the individual amino acids (same concentration as in AA+ medium). These media were adjusted to pH 7.5, filter-sterilized (0.2 mM) and supplemented with 10% dialyzed FBS (GIBCO) before use.

### Cell purification and culture

The PBMCs derived from healthy donors were isolated from peripheral blood by Ficoll-Hypaque gradient separation, CD8^+^ cells were purified by microbeads (BD Bioscience) followed by activation with anti-CD3 and anti-CD28 (Stemcell) for the indicated times.^27^ Mice lymphocytes were isolated from the spleen, CD8^+^ cells were purified by microbeads (BD Bioscience). Sorted cells were cultured in plates coated with anti-CD3 (2C11, 10 mg/mL) and anti-CD28 (37.51, 10 mg/mL; both from Biolegend) for the indicated times.

### Generation of knockdown cells

For generating interfering targeting the *Rraga*, *Rragb*, *Rragc* or *Rragd* OT-I CD8^+^ T cells, retrovirus was produced via triple transfection of HEK-293T cells with a retroviral transfer vector (pmKO.1-GFP for short hairpin RNA (shRNA)-*Rraga*, shRNA-*Rragb*, shRNA-*Rragc* and shRNA-*Rragd* construction) and the packaging plasmid pCL-Eco at a 1:1 ratio by using a polyethyleneimine-based DNA transfection reagent (Sigma-Aldrich), as recommended by the manufacturer. The viral supernatant was collected 48 hours after transfection, filtered through a 0.45 μm filter and stored at −80°C. OT-I CD8^+^ T cells were activated with plate bounded anti-CD3 (2C11, 10 mg/mL) and anti-CD28 (37.51, 10 mg/mL; both from Biolegend) for 48 hours, and then transduced with retroviruses as described above, supplemented with polybrene (8 µg/mL, Sigma-Aldrich) and centrifuged at 2000 rpm and 32°C for 1 hour. The transduced CD8^+^ T cells were fed with fresh medium for 48 hours, and the knockdown efficiency was confirmed by real-time PCR before adoptive transferring.

For generating *Slc3a2* knockdown cells, retrovirus were produced as described above. MC38 tumor cells were infected with the virus supernatant for 48 hours, and the supernatant was collected for CD8^+^ T cells culture. Knockdown efficiency was validated by flow cytometry.

### In vitro T-cell stimulation

For amino acid stimulation of freshly isolated naïve human or mice CD8^+^ T cells, the naïve T cells were sorted with Mouse Naïve CD8^+^ T Cell Isolation Kit (Stemcell), or Human Naïve CD8 T Cell Enrichment Set (BD Bioscience). The cells were washed and starved in AA− medium for 90 min at 37°C, followed by restimulation with AA−, AA+ or single amino acids medium for 30 min at 37°C.

For TCR crosslinking for freshly isolated naïve mice CD8^+^ T cells: naïve CD8^+^ T cells were sorted, followed by incubation with plate-bounded anti-CD3 (2C11, 10 mg/mL) and anti-CD28 (37.51, 10 mg/mL; both from Biolegend) in the presence of amino acids for indicated time. Cells were immediately spun down, washed once in cold phosphate-buffered saline (PBS) for analysis for phosphoflow staining.

For tumor supernatants culture, CD8^+^ T cells were cultured with fresh complete medium, tumor supernatants with or without different concentrations of LEU for 90 min and then activated with plate bounded anti-CD3 and anti-CD28 with indicated medium for 30 min. Cells were immediately spun down, washed once in cold PBS for analysis.

### Tumor inoculation and treatments

B16-OVA cells (2×10^5^) were injected subcutaneously into CD45.1 mice. At day 7, mice bearing tumor of a similar size were randomly divided into groups, sh-scrambled, sh-*Rraga*, sh-*Rragb*, sh-*Rragc* or sh-*Rragd* OT-I CD8^+^ T cells (5×10^6^) were injected intravenously, respectively. Mice were sacrificed after 14 days. MC38 cells (5×10^5^) were injected subcutaneously into *Cd4*^Cre^*Rragd^fl/fl^* or *Cd4*^Cre^ mice. MC38-OVA cells (5×10^5^) were injected subcutaneously into *Rag1*^−/−^ mice. Mice bearing tumor of a similar size were randomly divided into groups, *Cd4*^Cre^ *Rragd^fl/fl^* OT-I CD8^+^ T cells (2×10^6^) or *Cd4*^Cre^ OT-I CD8^+^ T cells (2×10^6^), *Cd4*^Cre^ *Rragd^fl/fl^* OT-I CD8^+^ T cells (1×10^6^) and *Cd4*^Cre^ OT-I CD45.1.2 CD8^+^ T cells (1×10^6^) (for co-transfer experiment), were injected intravenously. Mice were sacrificed at indicated days. Treatment of antiprogrammed cell death protein 1 (anti-PD-1) antibody (RMP1-14), or IgG isotype control (2A3) were given intraperitoneally at a dose of 100 µg per mouse on day 3 after tumor cell inoculation, then every 3 days for the duration of the experiment. LEU or PBS was given by intratumor injection at a dose of 70 mg/kg per mouse on day 3 after tumor inoculation, and every 2 days for the duration of the experiment. To isolate TILs, MC38 or MC38-OVA tumor were excised, minced and digested with 0.5 mg/mL collagenase IV (Sigma) and 200 IU/mL DNaseI (Sigma) for 30 min at 37°C, and then passed through 70 µm filters to remove undigested tumor tissues. TILs were then isolated by CD8 (TIL) MicroBeads (Miltenyi Biotec).

### Flow cytometry

For analysis of surface markers, cells were stained in PBS containing 0.5% (wt/vol) bovine serum albumin (BSA), with indicated antibodies. Surface proteins were stained for 30 min with the relevant fluorochrome-conjugated monoclonal antibodies in PBS containing 0.5% BSA on ice. The following antibodies were used: H-2Db Adpgk Neoepitope Tetramer (MBL, TB-5113-4), anti-CD8a (53-6.7), anti-CD69 (H1.2F3), anti-CD44 (IM7), anti-CD62L (MEL-14), anti-KLRG1 (2F1), anti-CD127 (A7R34), anti-LAG3 (C9B7W), anti-PD-1 (J43), anti-TIM3 (RMT3-23), anti-CD39 (Duha59), Intracellular RagD (ab187679, Abcam) was performed with a fixation/permeabilization kit (BD Biosciences). Ki-67 (SolA15; Thermo Fisher Scientific) were analyzed in cells fixed and permeabilized with Foxp3 staining buffers per the manufacturer’s instructions (Thermo Fisher Scientific).

For intracellular cytokine staining, cells were stimulated for 4 hours with phorbol myristate acetate and ionomycin in the presence of monensin (Thermo Scientific), then performed with a fixation/permeabilization kit (BD Biosciences). The antigen-specific intracellular cytokine staining was conducted as previously described.^28^ Cells were stimulated with OVA_257-264_ or MC38 Adpgk peptides, respectively.^29 30^ Cells were co-stimulated with 2 µg/mL anti-CD28 (Biolegend) for 1 hour, then incubated with 5 µg/mL brefeldin A (Topscience), 2 µM monensin (Thermo Scientific). After a total of 6 hours, cells performed with a fixation/permeabilization kit (BD Biosciences), further stained with interferon (IFN)-γ (XMG1.2), tumor necrosis factor (TNF)-α (MP6-XT22), interleukin (IL)-2 (JES6-5H4), granzyme B (GZMB) (QA18A28).

For phosphoflow staining, cells were fixed with 1× Lyse/Fix (BD Biosciences) buffer at 37°C for 10 min, washed and permeabilized by ice-cold Perm III buffer (BD Biosciences) on ice for 30 min, followed by staining with phospho-S6 (S235/236) or phospho-4E-BP1 (T37/46, both from Cell Signaling Technology) for 30 min at room temperature. The LIVE/DEAD Fixable Viability Dyes (Thermo Scientific) was used to exclude dead cells for subsequent phosphoflow analysis.

For mitochondrial staining, lymphocytes were incubated for 30 min at 37°C with 10 nM Mito Tracker Deep Green (Life Technologies) or 20 nM tetramethylrhodamine (TMRM) (Life Technologies) after staining surface markers.

### Immunofluorescence assay

Immunofluorescence assay was performed as previously described.^31^ CD8^+^ T cells were sorted and cultured for 24 hours in μ-slide chambered coverslips (Ibidi; 80826) coated with α-CD3 and α-CD28 (1 µg/mL). Cells were washed with PBS and fixed with 4% poly-formaldehyde in room temperature for 10 min, then permeabilized with 0.1% Saponin in PBS for 15 min and blocked with 5% BSA PBS for 30 min. Cells were incubated with primary antibodies at room temperature for 1 hour. After washing with 0.1% Tween-20 PBS for three times, cells were stained with secondary antibodies for 1 hour, and 4’,6-diamidino-2-phenylindole dihydrochloride for 5 min. Samples were scanned with Zeiss LSM880 confocal microscopy and analyzed with Imaris. Primary antibodies used in IF assay include α-lysosomal-associated membrane protein 1 (α-LAMP1) (Thermo Fisher Scientific; clone 1D4B), α-mTOR (Cell Signaling Technology; clone 7C10). Images were obtained with LSM880 confocal microscopy (Zeiss). Image analysis and quantification were performed with Imaris 8.4 software (Bitplane).

### Special diet treatments

C57BL/6 mice aged 6–8 weeks that were raised on standard chow diets were fed isocaloric diets containing complete L-amino acids (control) or diets deficient for L-LEU (Trophic Animal Feed High-Tech, China). The composition of other nutrients, vitamins and minerals was equivalent between these diets. After 2 weeks, mice were euthanized and T cell analyzed.

### Gene expression profiling

RNA was purified from CD8^+^ TILs isolated from *Rragd^+/+^Cd4*^cre^ (*Rragd^+/+^*) or *Rragd^fl/fl^Cd4*^cre^ (*Rragd^−/^*−) mice MC38 tumor. The RNA-seq analysis was conducted as previous reported.^32 33^ Gene Set Enrichment Analysis (GSEA) was performed and visualized by the R package clusterProfiler. The gene list was arranged by logFC (from high to low) between two groups and genes having a log2FC ≥1 but false discovery rate (FDR) >0.2 were excluded. Hallmark or canonical pathways (Broad Institute) were used as reference datasets for GSEA. GSEA was run under 10 000 permutations and the p value was adjusted by Benjamini-Hochberg method.

### Statistical analysis

Differences between two or more groups were analyzed by Student’s t-test or one-way analysis of variance followed by Tukey’s test. Statistical significance performed using GraphPad Prism V.6. Flow cytometry results were analyzed using FlowJo software (Tree Star). P value <0.05 indicates a statistically significance difference.

## Results

### RagD was required for CD8^+^ TIL function in the TME

**Figure 1:**
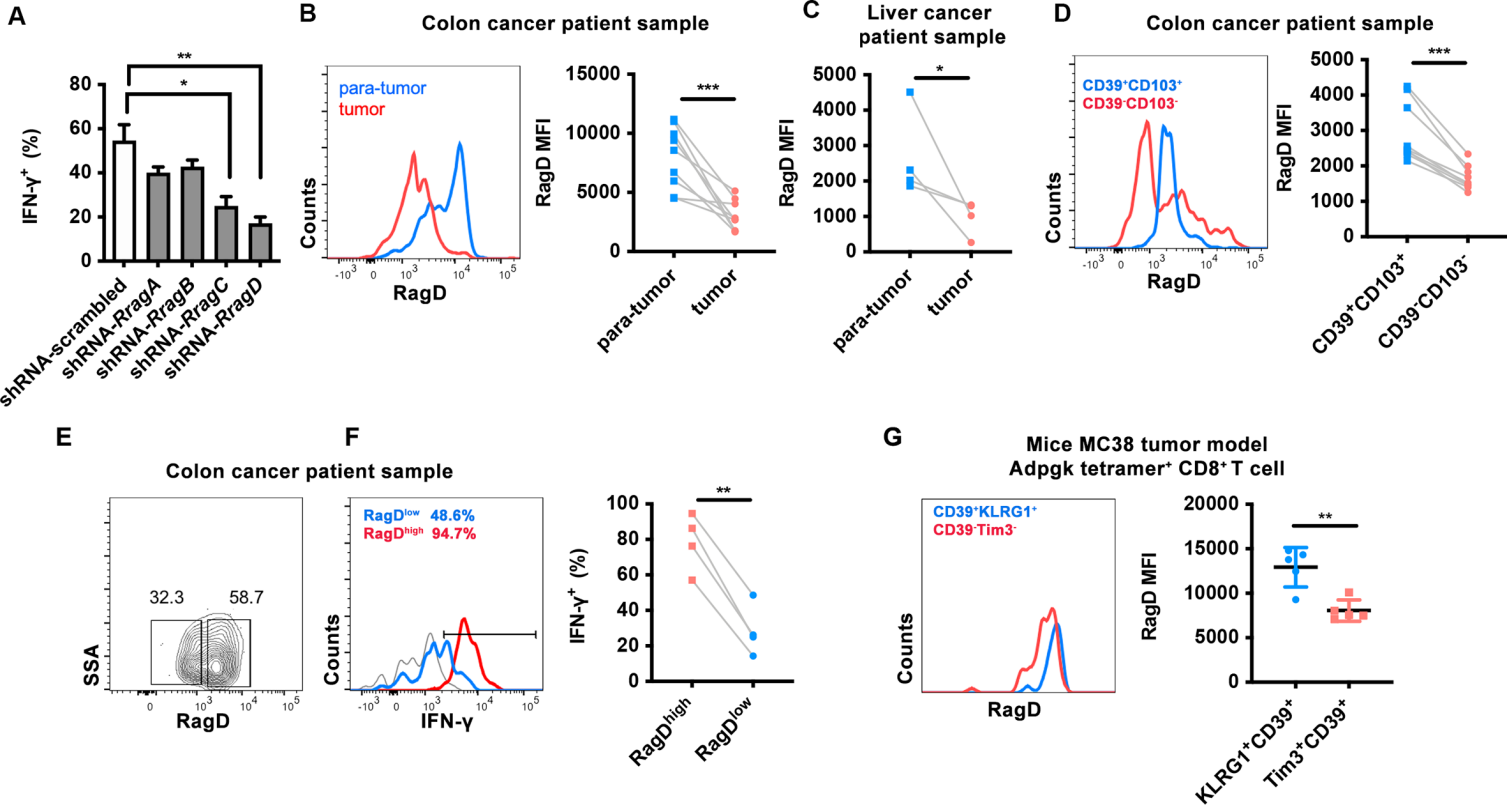
RagD are required for CD8^+^ tumor-infiltrating lymphocyte (TIL) functions in tumor microenvironment (TME). (A) OT-I CD8^+^ T cells were sorted and activated with plate bounded anti-CD3 and anti-CD28, followed by gene editing with short hairpin RNA (shRNA) targeting *Rraga*, *Rragb*, *Rragc* and *Rragd*. CD8^+^ T cells were transferred into CD45.1 mice bearing B16-OVA tumor. The intracellular cytokine staining for interferon (IFN)-γ on CD8^+^ TILs, on restimulation of phorbol myristate acetate (PMA) and ionomycin for 4 hours. The quantification of IFN-γ^+^CD8^+^ TILs (n=3). (B–C) Human CD8^+^ TILs isolated from patient’s tumor and para-tumor with colon cancer (n=7) and liver cancer (n=4). The RagD mean fluorescence intensity (MFI) was analyzed by flow cytometry. (D) Flow cytometry analysis for the correlation between RagD expression with CD39^+^CD103^+^ or CD39^-^CD103^-^ on CD8^+^ TILs from the patient with colon cancer (n=7). (E–F) Flow cytometry analysis for the correlation between frequencies of IFN-γ^+^ with RagD expression in CD8^+^ TILs from the patient with colon cancer (n=4). (F)The intracellular cytokine staining for IFN-γ on CD8^+^ TILs, on restimulation of PMA and ionomycin for 4 hours. Representative flow cytometry plots of RagD expression (E), IFN-γ expression (F), RagD^low^ (blue), RagD^high^ (red), unstimulated control (gray). (G) Flow cytometry analysis for the correlation between RagD expression with KLRG1^+^CD39^+^ or TIM3^+^CD39^+^ in H-2Db Adpgk Neoepitope Tetramer^+^ CD8^+^ TILs from mice MC38 tumor (n=5). Data are shown as mean±SD (error bars). Student’s t-test was used. *P<0.05; **p<0.01; ***p<0.001.

In order to dissect the molecular mechanisms of nutrient mTORC1 signaling in TILs, we focused on the evolutionarily conserved Rag family of small GTPases, which bridges amino acid sensing and mTORC1 activation.^34^ First, we examined whether Rag protein deficiency affected TIL function. First, CD8^+^ T cells were obtained from CD45.2 OTI mice and treated with or interfering shRNAs targeting *Rraga*, *Rragb*, *Rragc*, *Rragd* mRNAs or scrambled (shRNA-scrambled) (online supplemental figure S1A, B). We then adoptively transferred the cells into CD45.1 mice inoculated with B16-F10 melanoma cells expressing the cognate antigen (B16-OVA; online supplemental figure S1A, B). We found that the downregulation of Rag protein inhibited the cytotoxic activity of OT-I CD8^+^ T cells in vivo (figure 1A, online supplemental figure S1C). Among four Rag family members, RagD knockdown exerted the most suppressive effects on IFN-γ-secreting cells (figure 1A). Therefore, we speculated that RagD may be the most important factor for Rag GTPase activity in CD8^+^ TILs and merited further investigation.

10.1136/jitc-2020-002137.supp1 Supplementary data

To further explore whether RagD expression was associated with the effector functions of CD8^+^ TILs within the TME, we examined the expression of RagD in human CD8^+^ TILs extracted from patients with colon or liver cancer. Compared with CD8^+^ T cells in paratumor tissues, CD8^+^ T cells isolated from tumor tissues exhibited lower RagD expression levels (figure 1B, C). Furthermore, in colon tumor tissues, functional CD8^+^ TILs (CD103^+^CD39^+^) expressed higher levels of RagD (figure 1D).^35^ Moreover, RagD^low^ TILs lost the ability to secrete effector cytokines, such as IFN-γ (figure 1E-F). Additionally, in the MC38 tumor model, Tim3^+^CD39^+^ Tex cells exhibited lower RagD expression than KLRG1^+^CD39^+^ effector cells (figure 1G).^36^ Collectively, these findings demonstrated that CD8^+^ TILs exhibited decreased RagD expression in the TME. Since RagD expression was positively correlated with CD8^+^ TIL function, this protein could play indispensable roles in antitumor immune responses.

### RagD deficiency induced a dysfunctional phenotype in CD8^+^ TILs

**Figure 2:**
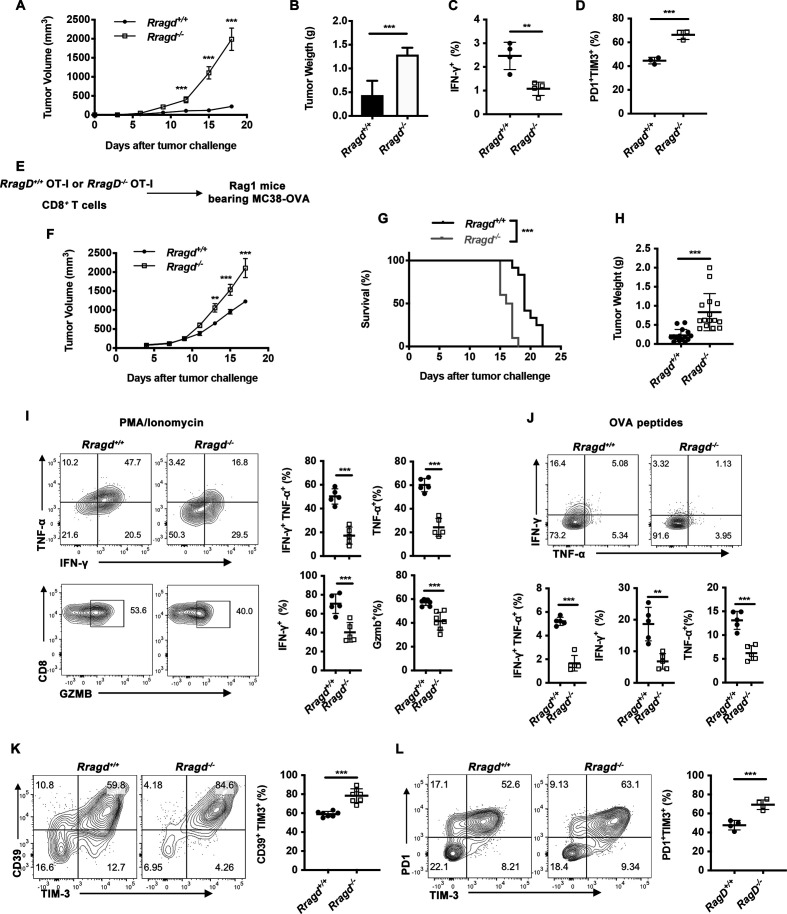
RagD-deficient CD8^+^ T cells show dysfunctional phenotypes. (A–D) MC38 mice tumor model on *Rragd^+/+^Cd4*^cre^ (*Rragd^+/+^*) or *Rragd^fl/fl^Cd4*^cre^ (*Rragd^−/−^*) mice. MC38 tumor growth kinetics (n=5) (A), tumor weights at end point (n=5) (B), quantification of interferon (IFN)-γ^+^ in CD8^+^ tumor-infiltrating lymphocytes (TILs) on restimulation of Adpgk peptides (n=3) (C), quantification of PD1^+^TIM3^+^ in CD8^+^ TILs (n=3) (D). (E–L) *Rragd^fl/fl^Cd4*^cre^ OT-I (*Rragd^−/−^*) CD8^+^ T cells or *Rragd^+/+^Cd4*^cre^ OT-I (*Rragd^+/+^*) CD8^+^ T cells were transferred into *Rag1*^−/−^ mice bearing MC38-OVA tumor. (E) Graphic of tumor model. (F) MC38-OVA tumor growth kinetics (n=10). (G) Mice survival (n=10). (H) Tumor weights of MC38-OVA tumor at 14 days after adoptive transfer (n=15). (I–J) At 14 days after adoptive transfer, intracellular cytokine staining for IFN-γ, tumor necrosis factor (TNF)-α and granzyme B (GZMB) on restimulation of phorbol myristate acetate (PMA) and ionomycin for 4 hours, or OVA_257-264_ for 6 hours (n=5). (K) At 14 days after adoptive transfer, quantification of CD39^+^TIM3^+^ in CD8^+^ TILs (n=7). (L) At 14 days after adoptive transfer, quantification of PD1^+^TIM3^+^ in CD8^+^ TILs (n=4). Data are shown as mean±SD (error bars) (B–D, G–L). Data are shown as mean±SEM (error bars) (A, F). Student’s t-test was used. **P<0.01; ***p<0.001.

To further investigate the in vivo functions of RagD in T cells, we crossed mice carrying floxed alleles for *Rragd* (*Rragd^fl/fl^*) with those expressing the *Cd4*-cre transgene (*Cd4*^cre^), herein designated as *Rragd^−/−^* mice. In these mice, RagD protein was specifically depleted in T cells. *Rragd^−/−^* mice appeared healthy at a young age and exhibited normal ratios of CD4^+^ and CD8^+^ T cells in the spleen (online supplemental figure S1D). Additionally, CD8^+^ T cells showed reductions in the CD44^+^CD62L^-^ effector-memory subpopulation, whereas CD4^+^ T cells displayed increased CD44^+^CD62L^+^ central memory subpopulation (online supplemental figure S1E). To examine the roles of RagD in regulating T-cell function, we investigated the growth of MC38 tumors in *Rragd^−/−^* mice. Compared with wild-type (WT) littermate controls, we found that MC38 tumors grew faster in *Rragd^−/−^* mice (figure 2A, B). Correspondingly, *Rragd^−/−^* mice showed a decrease in IFN-γ-secreting cells and an increase in the PD1^+^TIM3^+^ dysfunctional population from CD8^+^ TILs (figure 2C, D). To avoid any potential complications in the TME owing to the functions of Rag family proteins in dendritic cells or Tregs,^24 25^ we used an adoptive transfer system in which *Rragd^−/−^* OTI OVA-specific CD8^+^ T cells were adoptively transferred into T cell-deficient *Rag1*^−/−^ hosts bearing MC38-OVA tumors (figure 2E). Consistent with the MC38 tumor model (figure 2A, B), RagD-deficient antigen-specific CD8^+^ T cells failed to suppress tumor growth (figure 2F–H), as well as a decrease in the proportions of IFN-γ^+^ TNF-α^+^ polyfunctional and IFN-γ-secreting, TNF-α-secreting and GZMB-secreting subpopulations from CD8^+^ TILs (figure 2I, J). In addition, RagD deficiency elicited more CD39^+^TIM3^+^ or PD-1^+^TIM3^+^ dysfunctional TILs compared with WT controls (figure 2K, L).^36^ Alternatively, we also validated the effects of RagD deficiency on CD8^+^ T-cell dysfunction using an LCMV-Clone13 (Cl13) strain chronic infection mouse model (online supplemental figure S2A). On day 21 after infection, *Rragd^−/−^* P14 antigen-specific CD8^+^ T cells produced IFN-γ to a lesser extent than WT controls and elicited more CD39^+^TIM3^+^ or PD-1^+^TIM3^+^ dysfunctional populations (online supplemental figure S2B–D). Taken together, these data supported the important role of RagD in CD8^+^ T-cell antitumor immunity.

### RagD deficiency impaired cellular mTORC1 signaling and cell proliferation

**Figure 3:**
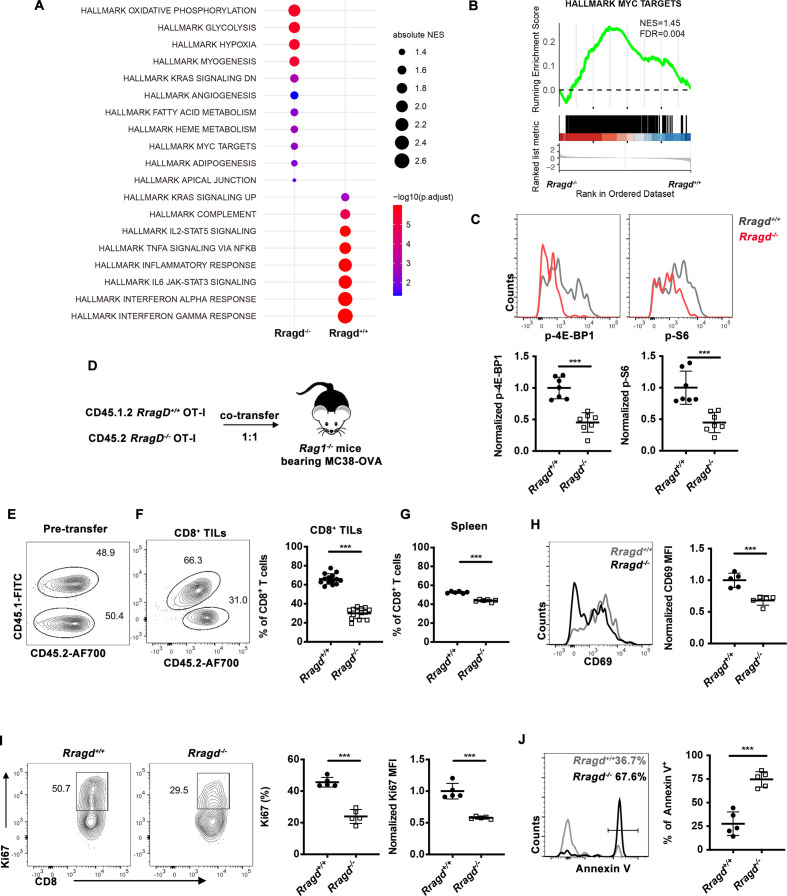
RagD deficiency inhibits the mammalian target of rapamycin complex 1 (mTORC1) activity of CD8^+^ T cells. (A–B) CD8^+^ tumor-infiltrating lymphocytes (TILs) from *Rragd^+/+^Cd4*^cre^ (*Rragd^+/+^*) or *Rragd^fl/fl^Cd4*^cre^ (*Rragd^−/−^*) mice bearing MC38 tumor. Gene set enrichment analysis (GSEA) identified upregulated and downregulated Hallmark pathways. (C) *Rragd^fl/fl^Cd4*^cre^ OT-I (*Rragd^−/−^*) CD8^+^ T cells or *Rragd^+/+^Cd4*^cre^ OT-I (*Rragd^+/+^*) CD8^+^ T cells were transferred into *Rag1*^−/−^ mice bearing MC38-OVA tumor. Expression of p-S6 or p-4E-BP1 on CD8^+^ TILs were stained and analyzed by flow cytometry (n=7). (D–J) *Rragd^fl/fl^Cd4*^cre^ OT-I (*Rragd^−/−^*) CD8^+^ T cells or *Rragd^+/+^Cd4*^cre^ OT-I CD45.1.2 (*Rragd^+/+^*) CD8^+^ T cells were mixed and co-transferred at 1:1 ratio into the same *Rag1*^−/−^ host bearing MC38-OVA tumor. (D) Graphic of tumor model. (E) The proportion of CD45.1 and CD45.1.2 cells in CD8^+^ T cells pretransfer. (F–G) Mice were analyzed at 14 days after adoptive transfer, the quantification of relative of CD45.1 and CD45.1.2 percentages in CD8^+^ T cells in the TILs (n=15) and spleen (n=5). (H) The mean fluorescence intensity (MFI) of CD69 on CD8^+^ TILs (n=5). (I) The quantification of frequencies and MFI of Ki67^+^ cells in CD8^+^ TILs (n=5). (J) The quantification of AnnexinV^+^ cells in CD8^+^ TILs (n=5). Data are shown as mean±SD (error bars). Student’s t-test was used. *P<0.05; **p<0.01; ***p<0.001.

In order to dissect the molecular mechanisms through which RagD regulated CD8^+^ T-cell functions within the TME, we isolated CD8^+^ TILs from MC38 tumors in *Rragd^−/−^* mice for transcriptome analysis. GSEA (FDR <0.05) of RagD-deficient CD8^+^ TILs showed distinct transcriptional and metabolic networks from the WT controls (figure 3A). In addition, the hallmark pathways of Myc targets were downregulated, suggesting impairment of mTOR signaling (figure 3B). Reductions in mTORC1 signaling were validated in RagD-deficient CD8^+^ T cells from both the MC38 tumor model and the Cl13 chronic infection model, as reflected by decreased phospho-S6 and phospho-4E-BP1 staining (figure 3C, online supplemental figure S2E, F).

Because mTOR complexes are critical regulators of cellular proliferation and survival, next we assessed the accumulation of CD8^+^ T cells on RagD-deficiency in the TME. To mitigate the effects of tumor heterogeneity in individual mice, we used a co-adoptive transfer system in which *Rragd^−/−^* CD45.2 OTI and *Rragd^+/+^* CD45.1.2 OTI CD8^+^ T cells were transferred at an equal ratio into the same MC38-OVA tumor-bearing *Rag1*^−/−^ hosts (figure 3D, E). Fourteen days after adoptive transfer, we found that the relative proportion of *Rragd^−/−^* OTI CD8^+^ T cells was markedly decreased in both spleen and tumor tissues (figure 3F, G), indicating the essential functions of RagD in T-cell expansion and survival. Reduced expression of the cell proliferation marker Ki67 and the activation marker CD69 was also observed in *Rragd^−/−^* OTI CD8^+^ TILs (figure 3H, I). Moreover, *Rragd^−/−^* OTI CD8^+^ TILs showed increased apoptosis (figure 3J). Taken together, our findings identified an intrinsic molecular mechanism in which RagD ablation in CD8^+^ T cells impaired cellular mTORC1 signaling, resulting in a defective metabolic program and reduced CD8^+^ T-cell proliferation and antitumor responses in the TME.

### RagD was essential for TCR-dependent mTORC1 signaling in CD8^+^ T cells

**Figure 4:**
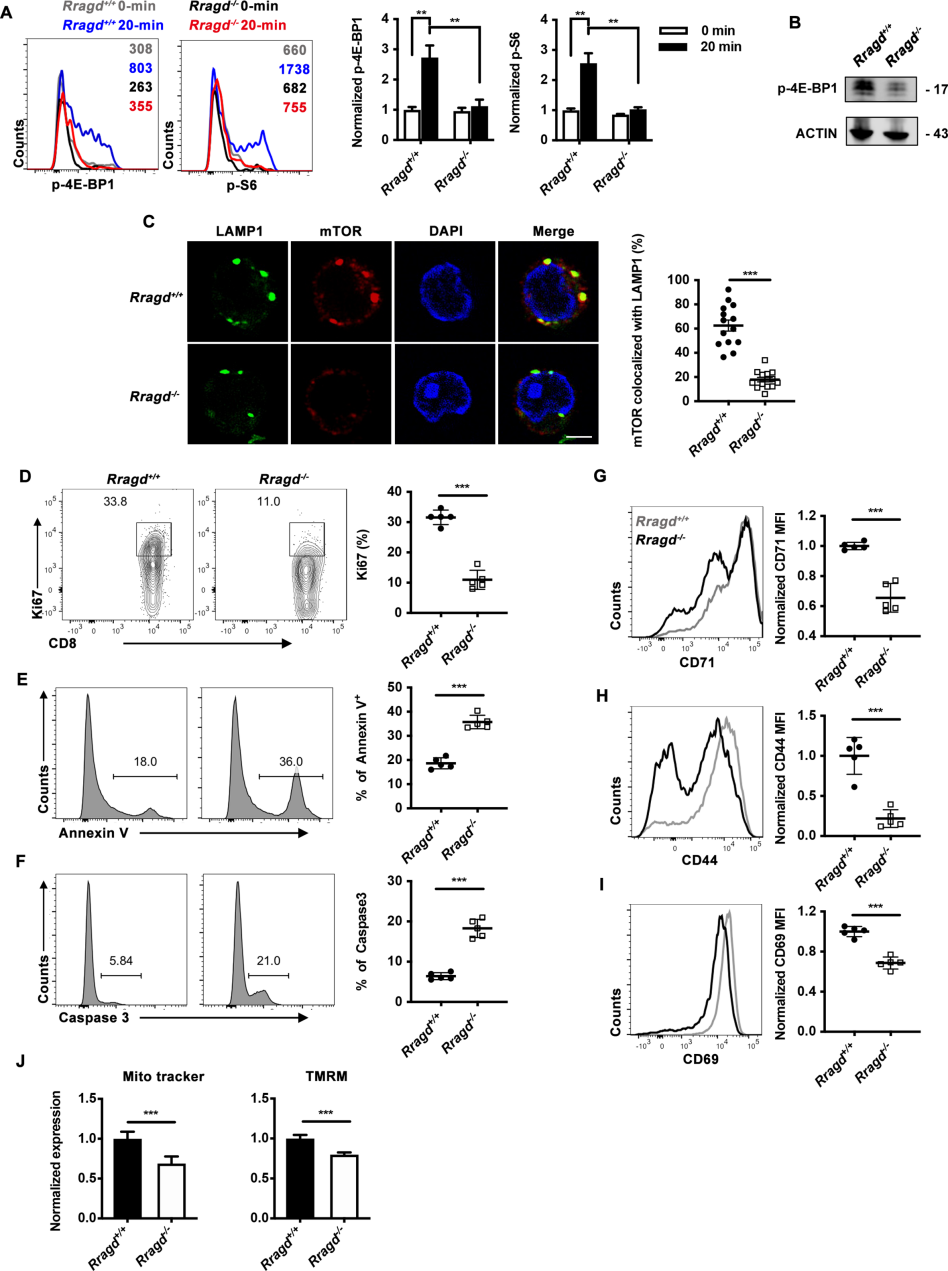
RagD deficiency restricted T-cell receptor (TCR)-induced mammalian target of rapamycin complex 1 (mTORC1) activity in CD8^+^ T cells. (A) Naïve CD8^+^ T cells from *Rragd^fl/fl^Cd4*^cre^ (*Rragd^−/−^*) or *Rragd^+/+^Cd4*^cre^ (*Rragd^+/+^*) spleen, stimulated with plate bounded anti-CD3 and anti-CD28 crosslinking. The expression of p-S6 or p-4E-BP1 were analyzed by flow cytometry, normalized to 0 min (n=5). (B) Immunoblot analysis of p-4E-BP1 levels in *Rragd^fl/fl^Cd4*^cre^ (*Rragd^−/−^*) or *Rragd^+/+^Cd4*^cre^ (*Rragd^+/+^*) CD8^+^ T cells stimulated with plate bounded anti-CD3 and anti-CD28 overnight (n=5). (C) Immunofluorescence staining of mTOR and lysosomal-associated membrane protein 1 (LAMP1) in naïve CD8^+^ T cells from *Rragd^fl/fl^Cd4*^cre^ (*Rragd^−/−^*) or *Rragd^+/+^Cd4*^cre^ (*Rragd^+/+^*) spleen stimulated with plate bounded anti-CD3 and anti-CD28 overnight (n=5). Scale bars, 5 μm. (D–J) Naïve CD8^+^ T cells from *Rragd^fl/fl^Cd4*^cre^ (*Rragd^−/−^*) or *Rragd^+/+^Cd4*^cre^ (*Rragd^+/+^*) spleen were sorted and stimulated with plate bounded anti-CD3 and anti-CD28 for 48 hours (n=5). (D) The quantification of Ki67^+^ cells in CD8^+^ T cells. (E) The quantification of AnnexinV^+^ cells in CD8^+^ T cells. (F) The quantification of Caspase3^+^ cells in CD8^+^ T cells. (G–I) The mean fluorescence intensity (MFI) of CD71, CD44 and CD69 on CD8^+^ T cells. (J) Quantification of MitoTracker and tetramethylrhodamine (TMRM) in CD8^+^ T cells. Data are shown as mean±SD (error bars). Student’s t-test was used. *P<0.05; **p<0.01; ***p<0.001.

TCR stimulation induces mTORC1 and CD8^+^ T-cell activation.^18^ Therefore, we further examined whether RagD deficiency affected TCR-mediated mTORC1 activation. We induced TCR signaling by treating naïve *Rragd^−/−^* CD8^+^ T cells with anti-CD3 and anti-CD28 antibodies (online supplemental figure S3A). WT control CD8^+^ T cells showed high mTORC1 activity after stimulation, whereas mTORC1 failed to receive TCR signals in *Rragd^−/−^*-naïve CD8^+^ T cells (figure 4A, B). To determine whether the impact of RagD deficiency was related to defects in the lysosomal translocation of mTORC1 in CD8^+^ T cells, naïve *Rragd^−/−^* CD8^+^ T cells were non-specifically activated, and colocalization of mTOR and the lysosome marker LAMP1 was assessed (figure 4C). Compared with naïve WT CD8^+^ T cells, RagD-deficient CD8^+^ T cells showed significant loss of mTORC1 recruitment to the lysosomal surface, indicating that RagD was essential for TCR-induced mTORC1 lysosomal translocation in CD8^+^ T cells (figure 4C). We further analyzed the downstream phenotypes of TCR-induced mTORC1 activation, including the proliferation and activation of CD8^+^ T cells. On TCR stimulation, reduced Ki67^+^ proliferating cells and increased AnnexinV^+^ and caspase 3^+^ apoptotic cells were observed in RagD-deficient CD8^+^ T cells (figure 4D–F). CD71, CD44 and CD69 expression levels were reduced in RagD-deficient CD8^+^ T cells (figure 4G–I). Finally, we analyzed mitochondrial fitness using MitoTracker for assessment of mitochondrial content and TMRM for mitochondrial membrane potential. Our results showed that staining for both markers was reduced in RagD-deficient CD8^+^ T cells (figure 4J). Taken together, these findings revealed the crucial functions of RagD proteins in promoting the translocation of mTORC1 to the lysosome and the maximal activation of this complex on TCR signaling, subsequently supporting the proliferation, activation and mitochondrial homeostasis of CD8^+^ T cells.

### Tumor cells limit T-cell access to LEU and impaired mTORC1 activity

**Figure 5:**
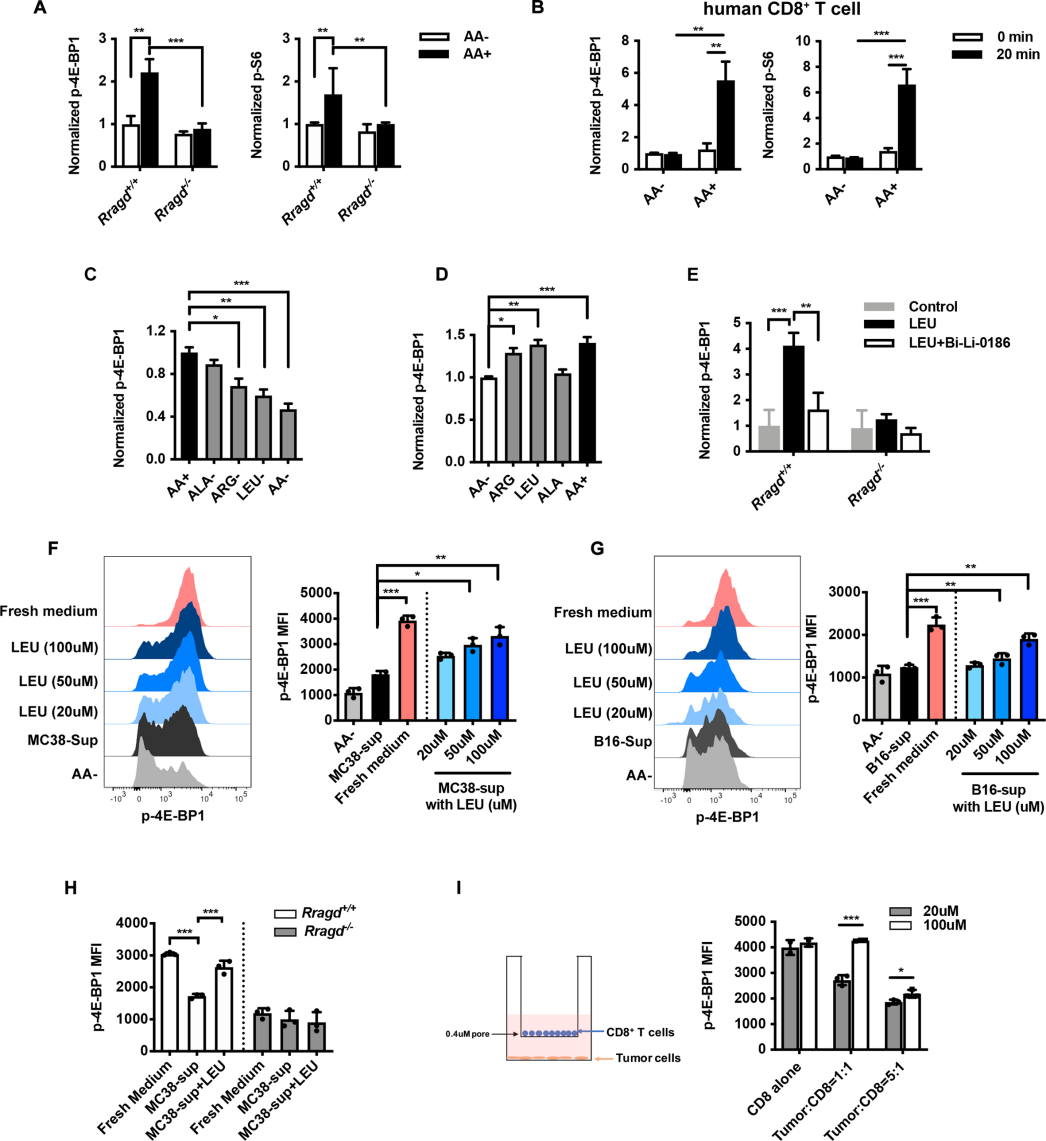
Tumor cells limited T-cell access to leucine (LEU) and impaired mammalian target of rapamycin complex 1 (mTORC1) activity. (A–B) Expression of p-S6 or p-4E-BP1 in naïve CD8^+^ T cells from *Rragd^fl/fl^Cd4*^cre^ (*Rragd^−/−^*) or *Rragd^+/+^Cd4*^cre^ (*Rragd^+/+^*) spleen (A), and healthy human donor (B), pretreated with or without amino acids, followed by anti-CD3 and anti-CD28 crosslinking in the presence or absence of amino acids (n=5). (C) CD8^+^ tumor-infiltrating lymphocytes (TILs) from MC38 tumor were stimulated with anti-CD3 and anti-CD28 in amino acid-deficient (AA−), amino acid-sufficient (AA+) or different single amino acid-deficient medium in vitro, p-4E-BP1 on CD8^+^ TILs was stained and analyzed by flow cytometry (n=5). (D) Naïve CD8^+^ T cells rested in AA− for 90 min and then stimulated in LEU, arginine (ARG) or alanine (ALA) medium for 30 min. p-4E-BP1 on CD8^+^ T cells was stained and analyzed by flow cytometry (n=5). (E) Naïve CD8^+^ T cells rested in AA− for 90 min and then stimulated in LEU medium for 30 min, in the presence or absence of RagD inhibitor Bi-Li-0186 (10 μM). p-4E-BP1 on CD8^+^ T cells was stained and analyzed by flow cytometry (n=5). (F–G) Effect of tumor cell culture supernatants on mTORC1 activity of CD8^+^ T cells. CD8^+^ T cells were rested with supernatants (sup) from cultured MC38 (F) or B16F10 (G) cells with varying concentrations of LEU, AA− or completed amino acid-sufficient fresh medium for 90 min and then stimulated with plate-bounded anti-CD3 and anti-CD28 for 30 min under the indicated conditions. p-4E-BP1 on CD8^+^ T cells was stained and analyzed by flow cytometry (n=5). (H) CD8^+^ T cells from *Rragd^fl/fl^Cd4*^cre^ (*Rragd^−/−^*) or *Rragd^+/+^Cd4*^cre^ (*Rragd^+/+^*) spleen were rested with sup from cultured MC38 with or without LEU (100 μM) for 90 min, followed by anti-CD3 and anti-CD28 stimulation for 30 min under the indicated conditions. p-4E-BP1 on CD8^+^ T cells were stained and analyzed by flow cytometry (n=3). (I) MC38 tumor cells and CD8^+^ T cells were cultured at different ratios for 48 hours in a Transwell system with 20 μM or 100 μM LEU. CD8^+^ T cells stimulated with anti-CD3 and anti-CD28 for 30 min, p-4E-BP1 on CD8^+^ T cells was stained and analyzed by flow cytometry (n=3). Data are shown as mean±SD (error bars). Student’s t-test was used. *P<0.05; **p<0.01; ***p<0.001.

Downstream of amino acids uptake, RagD activity is induced for mTORC1 activation in selective mammalian cells.^20^ To examine if amino acids affect TCR-induced mTORC1 activity, we pretreated naïve CD8^+^ T cells with amino acid-deficient or amino acid-sufficient medium and then crosslinked these with anti-CD3 and anti-CD28 antibodies. mTORC1 activity was higher in CD8^+^ T cells cultured with amino acid-sufficient medium than in those stimulated without amino acids in both human and mouse CD8^+^ T cells (figure 5A, B). Moreover, mTORC1 failed to receive TCR signals in *Rragd^−/−^*-naïve CD8^+^ T cells, regardless of amino acid supply, suggesting that amino acids and RagD were both indispensable for TCR-mediated mTORC1 activation (figure 5A).

To further identify which amino acids sustained mTORC1 activity in CD8^+^ TILs, we isolated CD8^+^ TILs from the MC38 tumor model and stimulated the cells with anti-CD3 and anti-CD28 antibodies in medium depleted of individual amino acids (online supplemental figure S3B). Among all examined amino acids, depletion of ARG or LEU most significantly impaired mTORC1 activity in CD8^+^ TILs (figure 5C). We recapitulated this finding by supplementing naïve CD8^+^ T cells with individual amino acids and found that ARG or LEU alone, particularly LEU, active 4E-BP1 phosphorylation (figure 5D). Next, to determine whether mTORC1 activation in CD8^+^ T cells by LEU was mediated by RagD, we used the pyrazolone compound BC-LI-0186 to specifically inhibit the interaction between RagD and the LEU sensor LRS.^22 37^ As expected, activation of mTORC1 by LEU was impaired by BC-LI-0186 in WT CD8^+^ T cells, whereas LEU failed to activate mTORC1 in *Rragd^−/−^* CD8^+^ T cells (figure 5E).

Finally, we tested whether tumor cells could impair mTORC1 activity in CD8^+^ T cells by altering LEU levels. Conditioned medium obtained from MC38 or B16F10 cells blocked phospho-4E-BP1 activation (figure 5F, G), whereas LEU supplementation in tumor cell conditioned medium restored phospho-4E-BP1 levels (figure 5F, G), although fresh medium with higher concentrations of LEU had minimal effect on 4E-BP1 phosphorylation in CD8^+^ T cells (Figure S3C). In addition, LEU supplementation failed to rescue mTORC1 activity in *Rragd^−/−^* CD8^+^ T cells (figure 5H). Moreover, we cultured MC38 and CD8^+^ T cells in a Transwell system (figure 5I). A high concentration of LEU (100 μM) had minimal effect on dampening mTORC1 activity in CD8^+^ T cells, whereas a low concentration of LEU (20 μM) decreased mTORC1 activity in CD8^+^ T cells, but not MC38 tumor cells (figure 5I, online supplemental figure S3D). Tumor glycolysis regulates T-cell functions; therefore, we cultured T cells in MC38 supernatants supplemented with glucose.^38^ However, the additional glucose failed to restore 4E-BP1 phosphorylation (online supplemental figure S3E). Taken together, these results supported the important roles of RagD and LEU in sustaining optimal mTORC1 activity in CD8^+^ T cells, and that tumor cells limited T-cell access to LEU, thereby impairing mTORC1 activity.

### LEU sustained CD8^+^ TIL antitumor immunity in vivo

**Figure 6:**
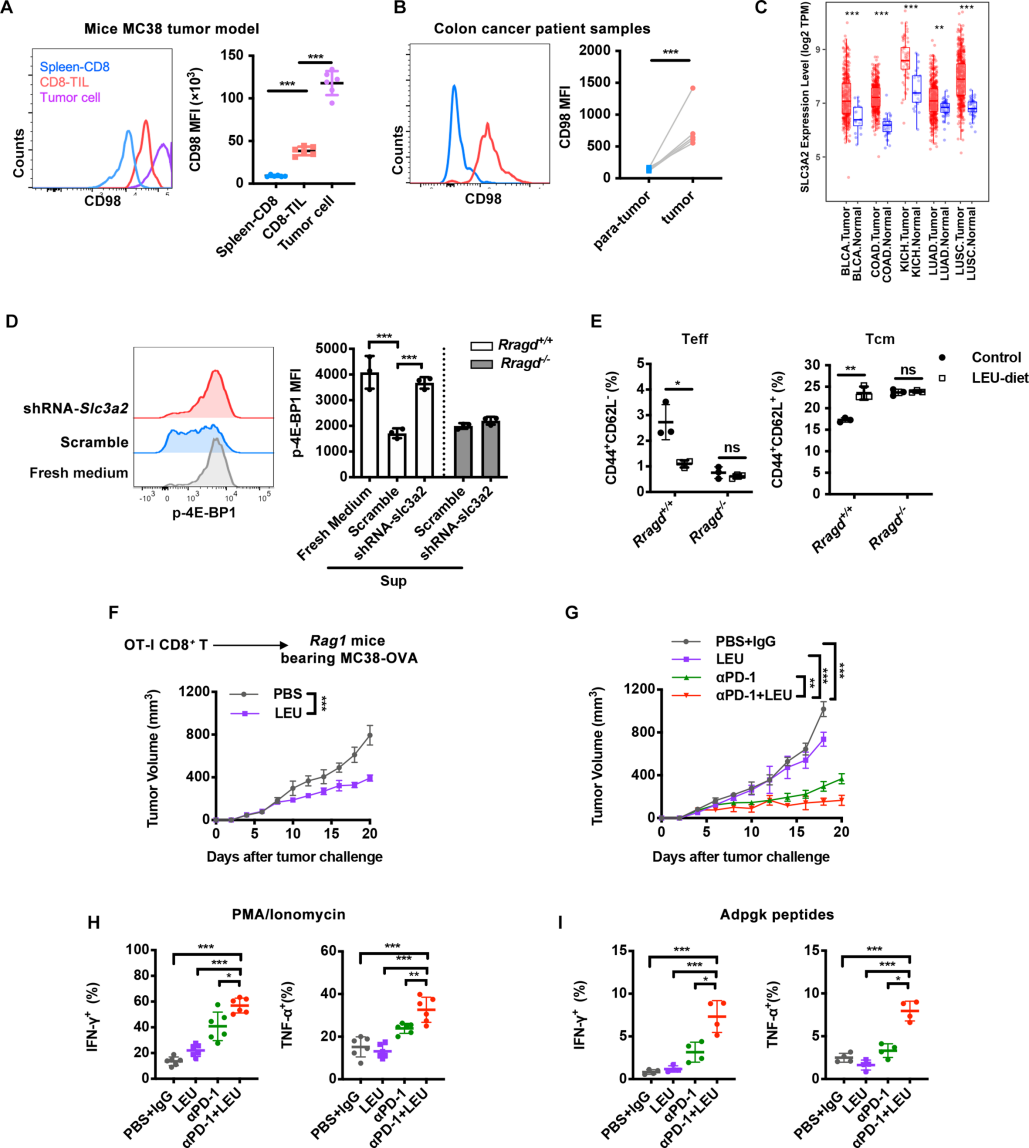
Leucine (LEU) sustained CD8^+^ tumor-infiltrating lymphocytes (TILs) immunity in vivo. (A) The mean fluorescence intensity (MFI) of CD98 on tetramer^+^CD8^+^ T cells from TILs or spleen, or on tumor cells in wild-type (WT) mice bearing MC38 tumor (n=6). (B) Human CD8^+^ TILs isolated from tumor and para-tumor with patient with colon cancer. The CD98 MFI was analyzed by flow cytometry (n=4). (C) Slc3a2 transcripts in tumors and paired adjacent normal tissue samples for several types of tumor from The Cancer Genome Atlas (TCGA). BLCA, bladder urothelial carcinoma; COAD, colon adenocarcinoma; KICH, kidney chromophobe; LUAD, lung adenocarcinoma; LUSC, lung squamous cell carcinoma. (D) Effect of supernatants (sup) from short hairpin RNA (shRNA)-*Slc3a2*-treated MC38 on mammalian target of rapamycin complex 1 (mTORC1) activity of CD8^+^ T cells. CD8^+^ T cells from *Rragd^fl/fl^Cd4*^cre^ (*Rragd^−/−^*) or *Rragd^+/+^Cd4*^cre^ (*Rragd^+/+^*) spleen were rested with sup for 90 min, followed by anti-CD3 and anti-CD28 stimulation for 30 min under the indicated conditions. p-4E-BP1 were stained and analyzed by flow cytometry (n=3). (E) Quantification of CD44 and CD62L expression on splenic CD8^+^ T cells of *Rragd^fl/fl^Cd4*^cre^ (*Rragd^−/−^*) or *Rragd^+/+^Cd4*^cre^ (*Rragd^+/+^*) fed complete L-amino acids (control), or leucine-deficient (LEU-diet) (n=3). (F) OT-I CD8^+^ T cells were transferred into the *Rag1*^−/−^ host bearing MC38-OVA tumor, LEU (70 mg/kg) or PBS was given by intratumor. MC38-OVA tumor growth kinetics (n=6). (G–I) Effect of combination of LEU (70 mg/kg) and antiprogrammed cell death protein 1 (αPD-1) treatment on WT mice bearing MC38 tumor. (G) MC38 tumor growth kinetics (n=6). (H) Intracellular cytokine staining for interferon (IFN)-γ and tumor necrosis factor (TNF)-α, on restimulation of phorbol myristate acetate (PMA) and ionomycin for 4 hours, or Adpgk peptides for 6 hours. The quantification of TNF-α^+^CD8^+^ TILs, IFN-γ^+^CD8^+^ TILs. Data are shown as mean±SD (error bars) (A–E, H–I). Data are shown as mean±SEM (error bars) (F, G). Representative plots, Student’s t-test were used. *P<0.05; **p<0.01; ***p<0.001.

In order demonstrate that tumor cells limited T-cell access to LEU in vivo, we evaluated the expression of CD98 (*slc3a2*), the heavy chain of the LEU transporter.^39^ Consistent with previous studies, CD98 expression was increased in CD8^+^ T cells on TCR activation (online supplemental figure S4A).^17^ Interestingly, CD98 expression was higher in antigen-specific CD8^+^ TILs than in splenic T cells isolated from the MC38 tumor model, indicating that CD8^+^ T cells were dependent on LEU to function in the TME (figure 6A). We further confirmed this finding with clinical colon cancer samples and showed that CD8^+^ T cells in colon tumor tissues exhibited higher CD98 expression compared with that in paratumor tissues (figure 6B). Then, we compared CD98 expression in CD8^+^ T cells and tumor cells in the MC38 tumor model and found that CD98 expression was significantly higher on tumor cells (figure 6A). Using data from The Cancer Genome Atlas (TCGA) database, we also found that CD98 transcript levels were higher in tumors than in matched normal tissues (figure 6C). The increased CD98 expression in tumor cells suggested its role in facilitating LEU uptake in competition with T cells within TME. Furthermore, we treated MC38 with scrambled or interfering shRNAs knocking down *Slc3a2* (online supplemental figure S4B). Then, we cultured CD8^+^ T cells with the supernatants from shRNA*-Slc3a2* cells or from tumor cells expressing scrambled shRNA. With only partially knockdown of CD98, CD8^+^ T cells cultured with supernatant from shRNA*-Slc3a2* cells showed significantly enhanced mTORC1 activity (figure 6D).

Next, we examined how LEU depletion affected the activity of CD8^+^ T cells in vivo. In mice fed LEU-deficient diets, splenic CD8^+^ T cells showed reductions in CD44^+^CD62L^-^ effector-memory subpopulation (Teff) and increased numbers of CD44^+^CD62L^-^ central memory subpopulation (Tcm), while the *Rragd^−/−^* mice did not response to LEU-deficient diets (figure 6E, online supplemental figure S4C). Moreover, we assessed whether the supplement of LEU in TME could restore CD8^+^ TIL function in vivo. Owing to the heterogeneity and complexity of the TME, we first employed a CD8^+^ T-cell adoptive transfer model. OTI CD8^+^ T cells were intravenously transferred to *Rag1*^−/−^ hosts after MC38-OVA tumor engraftment, the LEU was injected into tumor. LEU supplementation delayed tumor growth (figure 6F), but not enhanced tumor progression (online supplemental figure S4D), while the *Rragd^−/−^* OTI CD8^+^ T cells did not response to LEU-supplement (online supplemental figure S4E). Finally, we treated MC38 tumor-bearing mice with LEU with or without anti-PD-1 treatment (figure 6G). The combination of anti-PD-1 and LEU showed a synergistically inhibited tumor growth and enhanced cytokine production of CD8^+^ TIL (figure 6G–I, online supplemental figure S4F, G). Thus, these results supported that LEU plays important roles in sustaining CD8^+^ T-cell antitumor immunity in vivo, and the LEU-mTORC1 signaling pathway is RagD-dependent.

## Discussion

The TCR signaling pathway is required for T-cell activation and metabolic fitness.^14 40^ Nutrients also regulate the T-cell response; however, the mechanisms through which these nutrients affect the CD8^+^ T-cell program are still unclear. Here, we found that mTORC1 failed to receive TCR signals on amino acid insufficiency or in *Rragd^−/−^*-naïve CD8^+^ T cells, suggesting that amino acids and RagD were both indispensable for TCR-mediated mTORC1 activation. Despite this, it is still unclear whether RagD-mediated amino acid sensing represents a complementary pathway to TCR signaling or whether the T-cell dysfunction caused by overall metabolic suppression leads to inadequate mTOR activation. Our findings indicated that the Rag proteins, particularly RagD, acted as a quality control checkpoint for nutritional availability in order to ensure the presence of sufficient amino acids for mTORC1 activation and T-cell metabolic programs on TCR signaling initiation.

mTOR signaling is directly regulated through multiple metabolic processes in the T-cell response.^41^ However, because distinct T-cell activation states require different metabolic programs with their functional demands, the mechanisms regulating mTOR signaling in T cells during persistent antigen exposure are unclear.^42^ During chronic infection, antigen-specific CD8^+^ T cells exhibit attenuated mTOR signaling, even in the presence of antigen.^42^ PD-1 blockage partially restores mTOR activity, leading to enhanced expansion and function of cytotoxic T lymphocytes.^43 44^ Moreover, 7–8 days after chronic infection, T cells display bioenergetic insufficiencies, including mitochondrial dysfunction and impaired glycolysis.^45^ Rapamycin treatment improves the mitochondrial fitness of T cells.^45^ These reports collectively reveal the crucial roles of mTOR signaling in regulating T-cell function, primarily in mouse models of chronic infection. However, changes in bioenergetics differ between TILs and chronic infection, and the mechanisms through which mTOR signaling affects TIL function have not been fully elucidated.^45 46^ In this study, we identified the molecular mechanisms underlying the amino acid/mTORC1 signaling axis in CD8^+^ T cells within the TME. Our results suggesting that restriction of amino acid supply or low RagD expression may explain why CD8^+^ T cells lose mTOR expression, even with persistent antigen exposure, in the TME. Although further studies are still required to determine whether and how RagD expression is regulated in the TME, the correlation of RagD expression with T-cell dysfunction and the roles of RagD in amino acid sensing suggest potential therapeutic strategies based on RagD and its upstream manipulation.

Amino acids act as potent activators of mTORC1 signaling through multiple mechanisms.^47^ In this study, we found that mTORC1 activity was preferentially induced and sustained by LEU in CD8^+^ TILs. In CD8^+^ T cells, selectively depleting LEU impaired mTORC1 activity, similar to pan amino acid deprivation, and LEU alone was sufficient to restore mTOR activity in the absence of other exogenous amino acids. Notably, as substantial LEU is required for tumor cell proliferation, metabolism and mTORC1 activation, the effect of LEU restriction on tumor growth have been tested in vitro or in immune-deficient system.^48 49^ Here, our work demonstrated that sufficient LEU supply was indispensable for CD8^+^ T cells antitumor function within TME. Thus, targeting amino acid uptake and metabolism in tumor cells has been proposed to be a promising antitumor strategy,^50^ immune cells, particularly CD8^+^ TILs, are equally affected in the TME. Therefore, further studies are warranted to determine how immune cells, including CD8^+^ TILs, are affected by metabolic manipulation. Overall, our findings identified some new targets for cancer immunotherapy and established a platform for comprehensive evaluation of nutrient supplementation as a therapeutic approach in patients with cancer.

10.1136/jitc-2020-002137.supp2 Supplementary data

## Data Availability

Data are available in a public, open access repository. All data relevant to the study are included in the article or uploaded as supplementary information. RNA-seq datasets analyzed in the current study are available at the Sequence Read Archive (SRA) database (Accession number PRJNA704484, SRA number: SRR13774969, SRR13774970, SRR13774971, SRR13774972, SRR13774973, SRR13774974)
