# Supplementary material for: Amino acids and RagD potentiate mTORC1 activation in CD8+ T cells to confer antitumor immunity
Source: J Immunother Cancer. 2021 Apr 21;9(4):e002137. doi: 10.1136/jitc-2020-002137 (PMC8061841; doi:10.1136/jitc-2020-002137)
Supplement: Supplementary data [file jitc-2020-002137supp001.pdf]

Supplemental Methods and Materials

LCMV infection and calculation of viral loads

For clone 13 studies, 5–6-wk-old mice were infected i.v. with 4×10^6 PFUs LCMV clone 13 virus. For calculation of viral titers in serum, serum was collected from peripheral blood, and 10-fold dilutions were in- cubated on adherent Vero cells for 1 h. Cells were overlaid with a 1:1 mixture of medium and 1% agarose and cultured for 4 d. PFUs were counted after overlaying with a 1:1:20 mixture of medium: 1% agarose:neutral red for 16 h.

shRNAs

For The sequences for the shRNAs used are as follows:

shRNA-slc3a2: 5'-CCGGCCTTCTACAAAGTGCCAAGAACTCGAGTTCTTGGC ACTTTGTAGAAGGTTTTTG-3'; shRNA-RragA: 5'-CCGGCTTAGTATTGGACG CTATTTACTCGAGTAAATAGCGTCCAATACTAAGTTTTTG-3'; shRNA-RragB: 5'-CCGGGCAACAATACTAGACCGTATACTCGAGTATACGGTCTAGTATTGTT GCTTTTTG-3'; shRNA-RragC: 5'-CCGGCCTGTGGATATGCAGTCTTATCTCG AGATAAGACTGCATATCCACAGGTTTTTG-3'; shRNA-RragD: 5'-CCGGACA AGGACTCAACAGCCATTACTCGAGTAATGGCTGTTGAGTCCTTGTTTTTG-3'

Supplemental figures

Supplemental figure 1

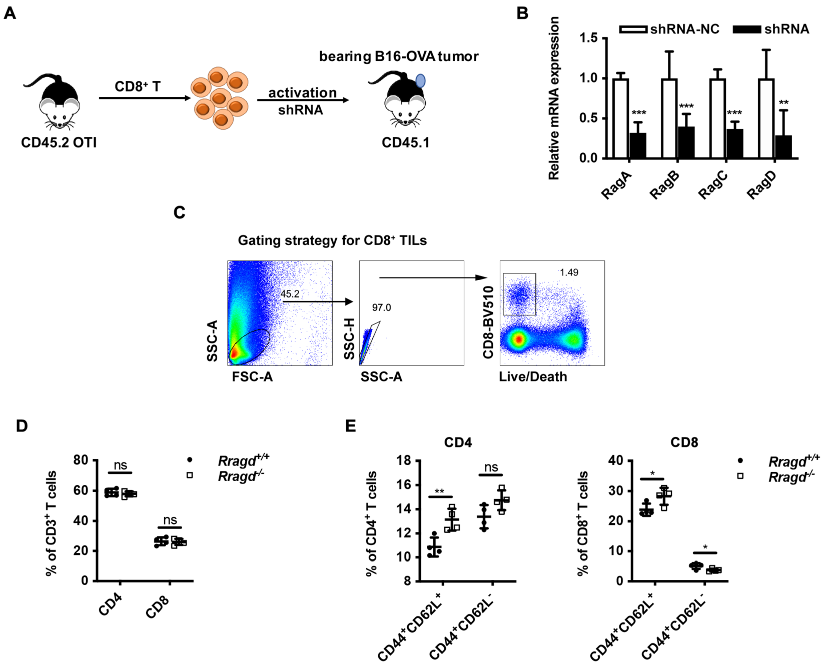

Supplemental figure 1. Loss of RagD impairs T cell anti-tumor immunity.
(A) Graphic of tumor model (n=3). OT-I CD8$^+$ T cells were sorted and activated with plate bounded anti-CD3 and anti-CD28, followed by gene editing with shRNA targeting *RragA*, *RragB*, *RragC*, and *RragD*. CD8$^+$ T cells were transferred into CD45.1 mice bearing B16-OVA tumor. **(B)** The realtime qRT-PCR showed shRNA knock down efficiency (n=5). **(C)** Gating strategy for FACS analysis of CD8 TILs used in this study. **(D)** Quantification of the frequency of splenic CD4$^+$ or CD8$^+$ T cells in 6-8-week-old *Rragd*$^{fl/fl}$*Cd4*^{cre} (*Rragd*$^{-/-}$) or *Rragd*$^{+/+}$*Cd4*^{cre} (*Rragd*$^{+/+}$) mice (n=4). **(E)** Quantification of the frequency of CD44 and CD62L expression on splenic CD4$^+$ or CD8$^+$ T cells in 6-8-week-old *Rragd*$^{fl/fl}$*Cd4*^{cre} (*Rragd*$^{-/-}$) or *Rragd*$^{+/+}$*Cd4*^{cre} (*Rragd*$^{+/+}$) spleen (n=4). Data were shown as mean ± SD (error bars). t test and was used. $P < 0.05$ indicates statistically significance difference. * indicates $P < 0.05$; ** indicates $P < 0.01$; *** indicates $P < 0.001$.

Supplemental figure 2

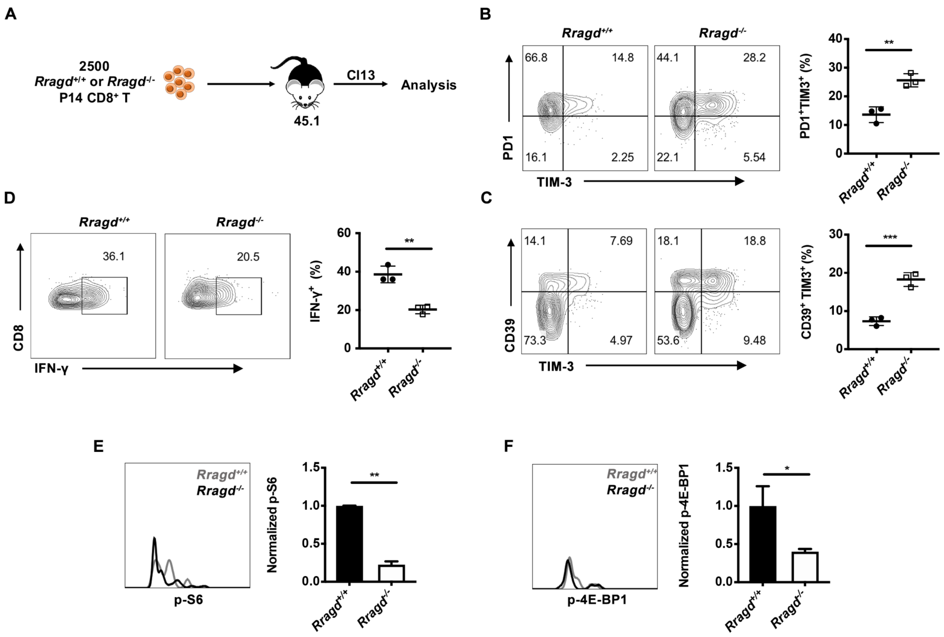

Supplemental figure 2. RagD deletion impaired CD8$^+$ T cell immunity in chronic infection. (A) Experimental design. 2.5×10^3 CD45.2 *Rragd*$^{fl/fl}$*Cd4*^{cre} P14 (*Rragd*$^{-/-}$), or CD45.2 *Rragd*$^{+/+}$*Cd4*^{cre} P14 (*Rragd*$^{+/+}$) CD8$^+$ T cells were adoptively transferred into CD45.1 mice and infected with Cl13. Spleens were analyzed 21 days later. **(B)** Quantification of CD39$^+$TIM3$^+$ in CD8$^+$ TILs (n=3). **(C)** Quantification of PD1$^+$TIM3$^+$ in CD8$^+$ TILs (n=3). **(D)** Splenocytes were re-stimulated with GP$_{33\text{-}41}$ peptides, and intracellular cytokine staining performed for IFN-γ. The quantification of IFN-γ$^+$ in CD8$^+$ T cells (n=3). **(E-F)** p-S6 or p-4E-BP1 on CD8$^+$ T cells were stained and analyzed by flow cytometry (n=3). Data were shown as mean ± SD (error bars). t test and was used. $P < 0.05$ indicates statistically significance difference. * indicates $P < 0.05$; ** indicates $P < 0.01$; *** indicates $P < 0.001$.

Supplemental figure 3

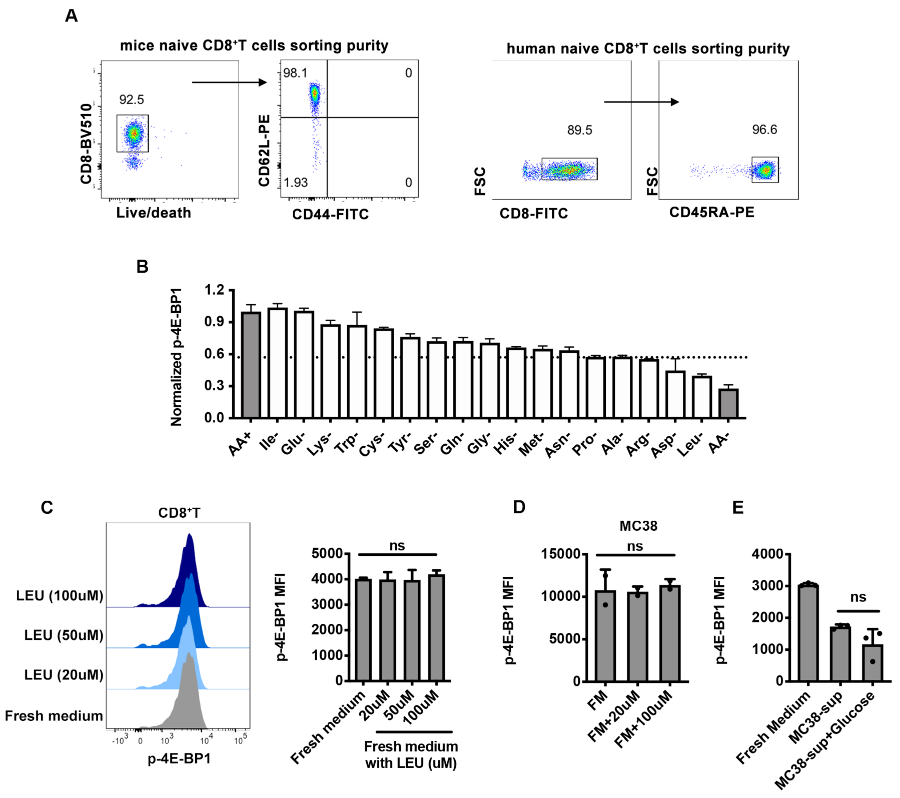

Supplemental figure 3. Leucine sustained mTORC1 activity in CD8⁺ TILs.
(A) flow cytometry analysis for the purity of naïve CD8⁺ T cells. **(B)** CD8⁺ TILs from MC38 tumor were stimulated with anti-CD3 and anti-CD28 in amino acid-deficient (AA-), amino acid-sufficient (AA+), or different single amino acid-deficient medium *in vitro*, p-4E-BP1 on CD8⁺ TILs was stained and analyzed by flow cytometry (n=3). **(C)** CD8⁺ T cells cultured with varying concentrations of LEU, and then stimulated with anti-CD3 and anti-CD28 for 30 min under the indicated conditions. p-4E-BP1 on CD8⁺ T cells was stained and analyzed by flow cytometry (n=3). **(D)** MC38 cultured with varying concentrations of LEU, and p-4E-BP1 was stained and analyzed by flow cytometry (n=3). **(E)** CD8⁺ T cells were rested with supernatants (sup) from cultured MC38 with or without glucose (2ug/mL) for 90 min, followed by anti-CD3 and anti-CD28 stimulation for 30 min under the indicated conditions. p-4E-BP1 CD8⁺ T cells was stained and analyzed by flow cytometry (n=3). Data were shown as mean ± SD (error bars). t test and was used. $P < 0.05$ indicates statistically significance difference. * indicates $P < 0.05$; ** indicates $P < 0.01$; *** indicates $P < 0.001$.

Supplemental figure 4

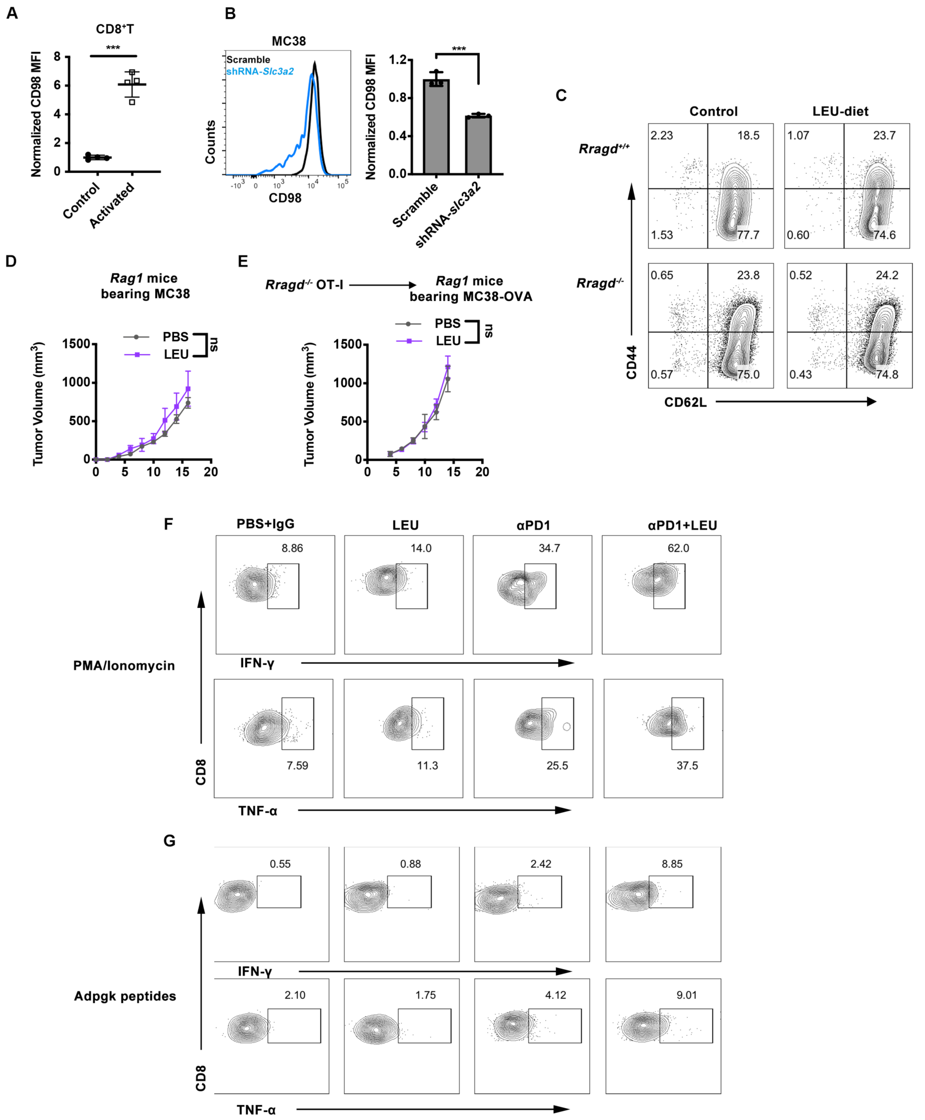

Supplemental figure 4. LEU sustained CD8+ TILs immunity *in vivo*
(A) Naïve CD8+ T cells were sorted and stimulated with anti-CD3 and anti-CD28 for 48 hours. MFI of CD98 was analyzed by flow cytometry (n=4). **(B)** The knock down efficiency of shRNA-*Slc3a2* on MC38 cell. **(C)** The frequency of CD44 and CD62L expression on splenic CD8+ T cells of *Rragd*$^{fl/fl}$*Cd4*^{cre} (*Rragd*$^{-/-}$) or *Rragd*$^{+/+}$*Cd4*^{cre} (*Rragd*$^{+/+}$) fed complete L-amino acids (Control), or leucine-deficient (LEU-diet) (n=3). **(D)** MC38 tumor growth kinetics in *Rag1*$^{-/-}$ host. LEU (70mg/kg) or PBS was given by intratumor (n=5). (**E**) *Rragd*$^{fl/fl}$*Cd4*^{cre} (*Rragd*$^{-/-}$) OT-I CD8+ T cells were transferred into the *Rag1*$^{-/-}$ host bearing MC38-OVA tumor, LEU (70mg/kg) or PBS

was given by intratumor. MC38-OVA tumor growth kinetics (n=5). **(F-G)** Effect of combination of LEU (70mg/kg) and anti-PD-1(αPD-1) treatment on WT mice bearing MC38 tumor. Intracellular cytokine staining for IFN-γ and TNF-α, upon restimulation of PMA and Ionomycin for 4h **(F)**, or Adpgk peptides for 6h **(G)**. Data were shown as mean ± SD (error bars). Representative plots, t test and was used. $P < 0.05$ indicates statistically significance difference. *** indicates $P < 0.001$.
