# Supplementary material for: Amino acids and RagD potentiate mTORC1 activation in CD8+ T cells to confer antitumor immunity
Source: J Immunother Cancer. 2021 Apr 21;9(4):e002137. doi: 10.1136/jitc-2020-002137 (PMC8061841; doi:10.1136/jitc-2020-002137)
Supplement: Supplementary data [file jitc-2020-002137supp002.pdf]

Amino Acids and RagD Potentiate mTORC1 Activation in $CD8^+$ T Cells to Confer Antitumor Immunity

Authors: Yiwen Zhang, Hongrong Hu, Weiwei Liu, Shumei Yan, Yuzhuang Li, Likai Tan, Yingshi Chen, Jun Liu, Zhilin Peng, Yaochang Yuan, Wenjing Huang, Fei Yu, Xin He, Bo Li*, and Hui Zhang*.

Graphical Abstract

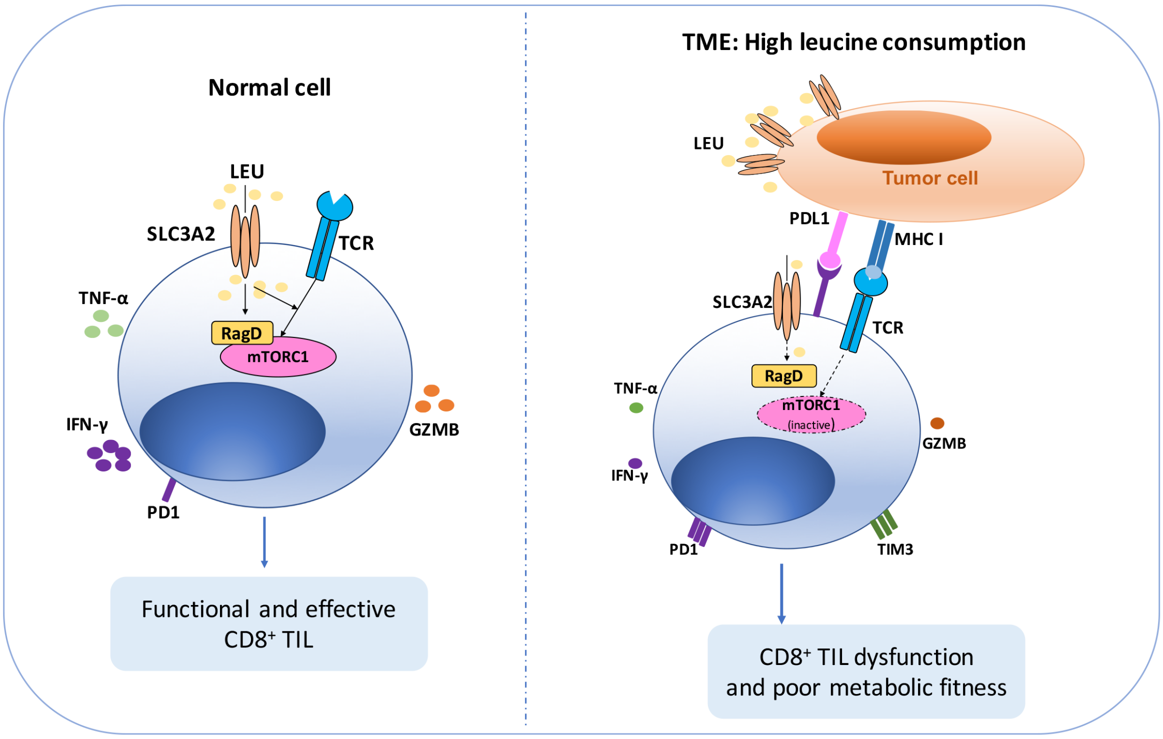

Bullet Points

Amino acid signals are regulated by RagD to potentiate TCR-mediated mTORC1 signaling in $CD8^+$ T cells

Abnormal LEU metabolism on mTORC1 activity in T cells and the TME contributes to $CD8^+$ T cell dysfunction

LEU supplementation improves T cell immunity in MC38 tumor-bearing mice in vivo
